# A theory of measuring natural selection and genetic monitoring

**DOI:** 10.1080/19420889.2022.2124631

**Published:** 2024-05-29

**Authors:** A. I. Yuriev

**Affiliations:** Biology, Museum Employee, Borissiak Paleontological Institute of the Russian Academy of Sciences, Moscow, Russia

**Keywords:** Population genetics, microevolution, genetic variability, gene flow, science funding

## Abstract

Two methods have been compared for determining the value of natural selection in the natural populations. The first method, based on the F_ST_-statistics, employs the dependence of genetic diversity of a species on the value of gene flow between subpopulations of the species, derived from the assumption that all the mutations are close to selective neutrality, and subpopulations effect each other equally. Susceptibility to selection is estimated by the degree of deviation from this relationship between genetic diversity and gene flow in certain species. The second method is based on the probability theory and involves comparison between stabilities of the forms, competing in the population, which is computed using the data about fluctuations in their occurrence in several generations. As applied to the problems of genetic monitoring of rare and valuable species, the first method can be employed for express-assessment of susceptibility of a species to rapid intraspecific changes. The second method is suitable for a long-term and in-depth genetic monitoring of the species subjected to extremely intense natural selection of a disruptive or stabilizing form, which were revealed using the first method. There is a lack of long-term observations of intraspecific genetic variation of rare and protected species. The need for funds that finance long-term genetic research is substantiated.

## Introduction

To date, conservation of genetic resources is an important goal. Thus, of interest is any method that makes it possible to assess the state of naturally occurring populations.

It is conceivable that any species of organisms has its unique genotype, since no combination of genes can appear twice. At that, in terms of the number of extinct species and species on the verge of extinction, the modern era can be compared with the epochs of major extinction events, similar to the extinction of dinosaurs. Numerous gene combinations disappeared and will disappear before the science will study their properties.

The problem is complex since, first, the gene pool is a conceptually variable object. The most powerful human influence on living populations cancels neither natural selection, nor other evolution factors. Rather on the contrary, an accelerated evolution should be expected, in an attempt to adapt to such conditions. Hence, we are dealing with the conservation of a variable object, and what is more, in an unstable situation.

Secondly, the conserved object has a certain stability and is able to adapt to the environment. If the environment worsens, the object cannot noticeably respond straight away. Hence, not in every instance the degree of threat can be readily determined.

Biologists-ecologists “in fact, examine the species, which alter right in their own backyard”, – Joel Braun from the University of Illinois in Chicago stated. They ignore it at their own risk and peril, since the efforts to conserve may lead to the evolution in an unexpected way, sometimes making the protected species unadapted to the environment over nearly several generations [1].

Hence, genetic monitoring of species has been on the agenda. Since there are numerous species, a question has to be posed as to exactly what kind of species must become the primary objects of genetic monitoring, and what parameters best suit its goals.

A whole number of authors explored this question, who proposed various parameters of populations for monitoring purposes: [2–7] and [8].

F.A. Aravanopoulos reviewed the suggested parameters to be monitored [9]. He concludes that natural selection, genetic drift, and gene flow, as essential forces on a microevolutionary scale, must be first and foremost evaluated for monitoring purposes.

To estimate natural selection, it is offered therewith to observe the temporal changes of three parameters: 1. Ratio between age-related and size classes. 2. Reproductive ability. 3. Number of plantlets per unit area (for plants). The two last parameters can be considered as those equivalent to the diagnostics of a life cycle, proposed by Schwartz, M.K., Luikart, G., and Waples, R.S [6].

Nevertheless, when making such an assessment, both practical and theoretical difficulties arise. Let us assume that, studying the classes of individuals, which compete in the population, we have expressed the ratios between their age-specific ranges, reproductive abilities, or some other parameters by some numbers. It is quite unobvious that this ratio characterizes exactly the natural selection, which is usually interpreted as a ratio between adaptivenesses.

Such research studies involve measurements of merely adaptiveness components (e.g., the same ratio between age-related ranges, survival rate prior to puberty, survival rate after environmental changes, number of partners or born descendants) as the substitute forms of general adaptiveness. An objection to it can always be raised, that all this fails to reflect natural selection, since the measured components of adaptiveness can differ within one generation or various years [10–13]. It is unclear, to what degree the selection, thus measured, is time-invariant.

That is exactly why we cannot use equations of classic population genetics to solve this problem. These deal with the monotone decrease and increase in the occurrence of the classes of individuals, which are not usually demonstrated during empirical observations. From the experiments made by P. L’Heritier and G. Teissier [14], to the recent publications, i.e., Siepielski AM, DiBattista JD, Carlson SM [12]., we have more frequently seen chaotic fluctuations rather than monotone changes in the occurrence of individual classes in the most diverse organisms.

It is clear that the changes in the occurrence of forms, caused by the difference in their adaptivenesses, gene drift, gene flow, and a founder principle, irrespective of the mechanism, act in the same manner: alter the incidence. Although these changes themselves are reversible and, strictly speaking, are not the selection, they can lead to withdrawal of some genotypes and establishment of others, what constitutes the act of natural selection, being an overall result of all these processes. Hence, natural selection monitoring must be aimed at defining the probability of a selection act, i.e., comparing the stabilities of each class of individuals.

Hence, our aim is to define the stability of a particular class of individuals in the population, with the knowledge of monitoring data for various classes of individuals. Whichever way we characterize the competing classes of individuals by age-related range, number of descendants, or otherwise, in any case, the data about the ratio between the occurrences of two or more classes of individuals inside the population, which varies in a number of generations, will be the initial material to determine the stability.

To estimate the stability of the classes of individuals, two approaches can be presented. Based on the data about the genetic structure of a species, an effort can be made to evaluate the significance of the F_ST_-statistics-based selection, using the fact that it relies on the “neutralist” model. The greater the observed result differs from that predicted by the model, the farther the situation in the population is from the selective neutrality of the classes of individuals. I.e., the more the studied classes of individuals are subjected to the natural selection. The second approach implies that, the monitoring data over several generations enable a comparison of the classes of individuals in terms of stability using the probability theory, having evaluated the stability of each of them, based on the characteristic of their fluctuations.

Since there are many species of organisms, of importance is the question, which of them must become a primary object of genetic monitoring. Aravanopoulos thinks that genetic monitoring must be focused on 1. key species of perennial plants of biological and economic importance, starting from ecologically dominating species (to prevent), and 2. rare/endangered species (to restore). One can agree with this, with the reservation that, there are many both species of biological importance, and rare species. By no means, all of them are under uncontrolled alteration threat. Among them, in turn, a ”risk group” must be specified, for which the modern science has all the required criteria.

Typically, in the conservation-oriented research, a F_ST_-statistics has been employed as a method to evaluate the ability of a species to preserve the genetic unity. With its help, though with some assumptions, the value of the gene flow between subpopulations of a species can be computed. However, it is not always clear, whether a large, calculated gene flow really means that subpopulations intensely exchange individuals or genetic material.

Since the value of the calculated gene flow constitutes just a function of similarity and difference between subpopulations by the incidence of genes, it can be interpreted as an indicator of subpopulations’ individualization recentness. The interpretation depends on the presupposition, whether we consider the species history or the existing interpopulation relationships as a creator of the studied genetic structure.

However, it can be concluded that the species with too poor calculated gene flow is doomed to fragmentation, irrespective of whether it is caused by remote individualization of subpopulations or insignificant current exchange between them. Hence, such a species is under threat and requires monitoring. It can also be said that the species with a very large, calculated gene flow will be subjected to outbreeding depression in some subpopulations due to plenty of individuals, inadaptive in the specific local conditions. It is possible for both the intense exchange, and the recent individualization of its subpopulations.

It can be concluded that the species with both very large, and very poor calculated gene flow must be a key object of genetic monitoring. However, the F_ST_-statistics relies on the island model of S. Wright, which proceeds from a rather big assumption about fairly modest effect of the natural selection value on the genetic structure of a species.

S. Wright, the model’s author himself, calls it the “simplest” one. The incidences of alleles in subpopulations in this model are distributed depending on the “systematic pressure of the environment”, which is caused by mutation process, gene flow, and natural selection [15]. It assumes without stating it that the level of selective differences between subpopulations is rather high to create the visible differences between them in the occurrence of genes, though too low to disguise the effect of the gene material inflow from other populations on these differences. It has been this situation that allows for computing F_ST_ and gene flow.

Undoubtedly, such an assumption is close to the truth in many cases; however, it is not to be expected that it will always be valid. Consideration of any taxon indicates that both slowly, and rapidly evolving groups exist. It should be expected that there is a considerable diversity in the intensity of natural selection in the populations. Hence, a class of situations exists, when no adequate result can be achieved employing the F_ST_-statistics.

If all the mutations had been selectively neutral, mutant individuals would have accumulated equally in all the subpopulations of a species, since both their emergence, and incoming with the flow of genes, as well as elimination, in each subpopulation would have had equal incidences. It is unclear, whence the differences in the incidences of genes and phenes between subpopulations would have appeared. For these to exist, some trend must be assumed in the selective differences between subpopulations, i.e., “centrifugal” forces, aiming to increase intersubpopulation differences, or to be more accurate, the differences between subpopulations subjected to a founder principle, gene drift, or natural selection, to which exactly a real flow of the genetic materials between subpopulations withstands, as a “centripetal” force.

Then, not only the species with the very large, and the very poor gene flow must be of interest for genetic monitoring, but also the species with the very strong or too downward trend of selective differences between subpopulations.

Assume that the selective differences between subpopulations are far greater than the effect of the exchange of genetic material between them. Then, the gene flow value provided by the F_ST_-statistics, will be vastly understated, as compared to the real number of interpopulation crossings. But if the selective differences between them are far slighter than the effect of the exchange of genetic material, the value of gene flow will conversely be overstated. In this case, the effect of the trend in selective differences can be expressed in terms of quantity as the value of error, when computing the gene flow. So, its value can be evaluated, upon learning how to measure the value of error, when employing the F_ST_-statistics. For this purpose, we will use a refined island model.

## A method for evaluating the effect of F_ST_-statistics-based selection

Let us take as a basis the Wright’s island model, though supplementing it with such a provision: mutations occur in all subpopulations with equal incidence, then they are distributed by the gene flow identically in all the subpopulations, where they remain for some time in the form of balanced polymorphism, and later are fixed or eliminated by natural selection.

Since molecular biology shows that in actual life all the populations are full of mutations, it should be supposed that the rates of emergence, distribution, and disappearance of mutations are similar. Then, if the gene flow is very large, then any sample taken from any subpopulation, will provide us with all or almost all the diversity of mutations, existing across macropopulation as a whole. If the gene flow is very poor, the sample will bring us only those mutations, which emerged in one or several subpopulations.

Then, there should exist a unique dependence of the gene flow value on intrapopulation diversity, e.g., the percent of polymorphic loci.

**Figure 1.**
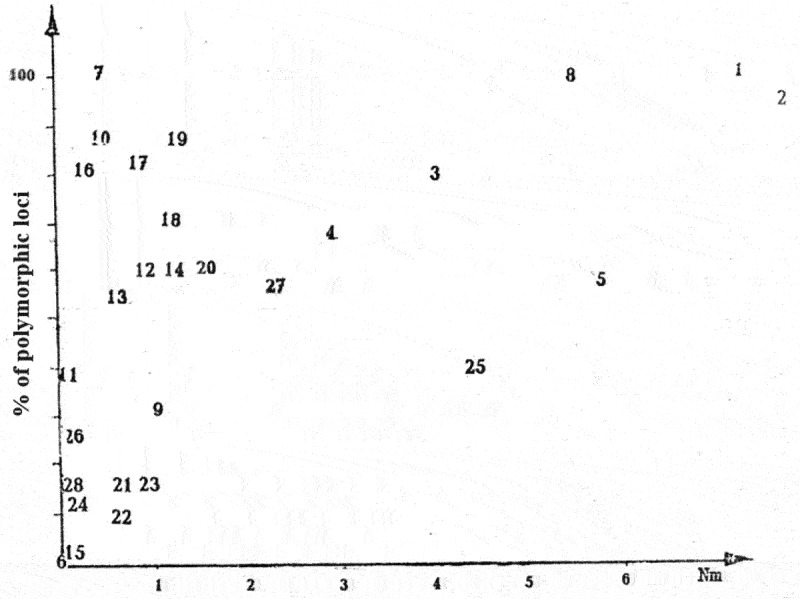
Dependence of genetic diversity on the flow of genes. 1. Pinus silvestris. 2. P. rigida. 3. P. longaeva. 4. P. ponderosa. 5. P. sibirica. 6. P. torreyana. 7. Eucalyptus obliqua. 6. E. pauciflora. 9. E. cloesiana. 10. E. delegatensis. 11. E. caesia. 12. Clarkia biloba. 13. C. lingulata. 14. C. rubicunda. 15. C. franciscana. 16. Lupinus subcarnosus. 17. L. texensis. 18. Hymenopappus artemisifolius. 19. Baptisia leucophaea. 20. B. sphaerocapra. 21. Phlox drummondii. 22. P. cuspidata. 23. P. roemariana. 24. Oenothera biennis. 25. O. parviflora. 26. Avena barbata. 27. Stephanomeria exigua. 28. S. malheurensis. Nm is given mostly according to Govindaraju [17], and Goncharenko, Padutov, Silin [18], as well. Hamrick, Linhart, Mitton [19]: percent of polymorphic loci

Let us employ the graph of dependence of the polymorphic loci percent on the value of gene flow as exemplified by higher plants Figure 1 [16].

Indeed, a tendency is visible on the graph for the percent of polymorphic loci to increase and for the gene flow to rise from one species to another. Suppose that the percent of polymorphic loci depends only on the gene flow, and all other factors serve as a source of the observed deviation from such a dependence.

Based on the linear character of the dependence, we shall apply the least-square method and obtain the following equation:

P = 48.45 + 6.48 Nm;

where Р – a percent of polymorphic loci, discovered in the species, Nm – a gene flow between subpopulations of the species.

If the various species had differed only in the value of gene flow between subpopulations, this equation would have described an increase in the genetic diversity from species to species as the gene flow rises. In this case, all the species on the empiric graph in Figure 1 would have formed up along the regression line.

## The F_ST_-method results

However, as is seen in Figure 1, by no means all the points form up along the line. A considerable scatter is observed. Hence, the populations of actually existing species differ in not only the number of interpopulation migrating individuals. The frequency of mutations for the most studied species is identical, namely, about one mutation over generation per a thousand of individuals. Hence, the differences between the species have really been determined by an individual-specific situation with fixation and extinction of mutants.

The species of higher plants proved to be rather diverse in this regard. Significant distinctions have been seen even between the species of one genus, for instance, within the Baptisia or Eucalyptus genera. In other genera, e.g., in the Flox genus, the species are close to one another. No genetic diversity was identified in such rare endemic species as Сlarkia franciscana and Pinus torreyana, represented by few individuals.

## Discussion and interpretation of the F_ST_-method results

Hence, the situation in subpopulations of the species, adequate for the model, can be presented only as an equilibrium between “centrifugal” and “centripetal” forces, i.e., subpopulations that increase and decrease differences. A “centripetal” force is a flow of diaspores. We have empirically defined it as a calculated gene flow, which for all the subpopulations of a species is almost identical, and “centrifugal” forces involve a founder principle, gene drift, and natural selection. An actual flow of diaspores uniformly dispels mutations in all the subpopulations of a species, though their extinction and fixation in all the populations occurs unevenly, in one place faster, in another – slower. Hence, a case is possible, when the calculated gene flow is defined only by the number of migrating diaspores.

It may have been true that in some subpopulations of a species, the “centrifugal” forces balance the same in the others. I.e., in some subpopulations, mutations have occurred and have been maintained there by “centrifugal” forces, aiming to be fixed. In other subpopulations “centrifugal” forces aim to eliminate these mutations. But the flow of diaspores between subpopulations balances these processes. In this case, indeed, the greater the actual flow of diaspores is, the smaller the differences in the occurrence of mutations are. This is exactly the area of applicability of the Wright’s formula for a gene flow. Since the real species and intraspecific variations have ecological optimum and pessimum, a case of the selection, differently directed in various subpopulations, must occur frequently in the nature. It has been the species close to such an equilibrium that formed up along the regression line on graph 2.

Presuming that “centrifugal” factors in the subpopulations of a species are unbalanced, e.g., the pressure of selection or gene drift in the area of some subpopulations does not balance the same in others, or there are many pioneer subpopulations with a big role of the founder principle, then this is the case when the species is subjected to some microevolutionary changes. In such cases, the gene flow, computed using F_ST_, will prove to be understated as compared to the real number of intersubpopulation crossings, and it is likely to be relevant indeed to some species on graph 2.

What can cause the exceedance of the empiric genetic diversity over the theoretical one? If a species demonstrates far greater polymorphism, than the gene flow provides, it means that the content of polymorphic loci in its populations increases due to some other factor. A gene drift and a founder principle can only decrease polymorphism. Hence, quite a Darwinian natural selection, maintaining diversity, i.e., disruptive selection exists in these species. This one, as we have already known, can be balanced, or unbalanced between subpopulations.

Similarly, if the genetic diversity is far lower than the theoretical, this suggests that a factor affects the species, which decreases the percent of polymorphic loci. In case of the species, which incorporates small, long-isolated subpopulations, an intense gene drift can be supposed. A founder principle, in its turn, would have been able to play the same role in the quick-spread young species. However, if the populations are normal-sized, and it is difficult to assume rapid propagation, the depletion must be considered a result of intense stabilizing natural selection.

Hence, the value of natural selection for the species, at least in those subpopulations, where the samples were taken for a percent of polymorphic loci, can be conditionally determined as the difference between the empiric level of polymorphism and the level, which is defined by the above-mentioned formula, as the gene flow function:

S emp = Р emp – Р Nm.

**Figure 2.**
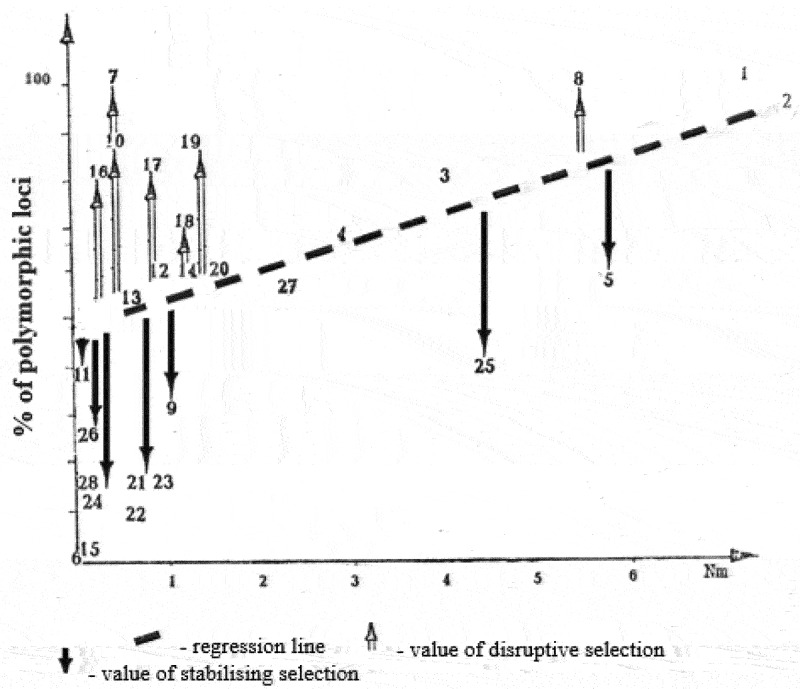
Dependence of genetic diversity on the value of gene flow. 1. Pinus silvestris. 2. P. rigida. 3. P. longaeva. 4. P. ponderosa. 5. P. sibirica. 6. P. torreyana. 7. Eucalyptus obliqua. 6. E. pauciflora. 9. E. cloesiana. 10. E. delegatensis. 11. E. caesia. 12. Clarkia biloba. 13. C. lingulata. 14. C. rubicunda. 15. C. franciscana. 16. Lupinus subcarnosus. 17. L. texensis. 18. Hymenopappus artemisifolius. 19. Baptisia leucophaea. 20. B. sphaerocapra. 21. Phlox drummondii. 22. P. cuspidata. 23. P. roemariana. 24. Oenothera biennis. 25. O. parviflora. 26. Avena barbata. 27. Stephanomeria exigua. 28. S. malheurensis. Nm is given mostly according to Govindaraju [17], and Goncharenko, Padutov, Silin [18], as well. Hamrick, Linhart, Mitton [19]: percent of polymorphic loci

It is clear that, generally speaking, not only the Darwinian selection but also a gene drift, and a founder principle will contribute here. However, in Figure 2, we do not see an obvious dominance of the process related to the genetic depletion of species over the process of enrichment, which would have been noticeable in the case of a considerable role of gene drift and founder principle. In Figure 2, white arrows show the value and direction of disruptive, and black – stabilizing selection for the different species of plants. The first one, respectively, is designated by positive values, and the second one – by negative ones. Apparently, the disruptive selection starts to have an impact, when the stabilizing one weakens in such a way that it can be presented in the form of a continuous scale of decreasing stabilizing selection with an increase, associated with it, in the disruptive one.

E.g., in five species of eucalyptus, presented in Figure 1 and Figure 2, we observe a considerable disruptive selection in the widespread species E. pauciflora and E.delegatensis, in which 3 and 2 subspecies, respectively, are distinguished. The same is seen in E. оbliquа, rather common as well, but confined to the limited conditions of moist mountain forests, it has 4 subspecies. Rare E. caesia, growing in small populations at the granite outcrops in southwestern Australia, turned out to be almost on the regression line, its conditions are close to “neutralist”, i.e., there is almost no selection, that enhances diversity, however, two subspecies can be distinguished in it. Finally, a local endemic species of the Queensland state E. cloesiana, rather widely, though sparsely spread in it, has proved to have a considerable stabilizing selection. As it should be expected, it has no subspecies [20].

A major dogma of the population genetics: “the richer the diversity, the higher the survival rate”, has come as a surprise: the more intense the stabilizing selection, the more endemic the species.

With all the approximate nature of such calculations, this is the way to get an idea about the values of “centrifugal” forces in the species with balanced and unbalanced selection. As the knowledge about population genetics of organisms accumulates, the graph in Figure 2 will be supplemented and revised, and the method will become more precise.

If we would like to express the species position on a “disruptive-stabilising” scale of selection by a specific number, we will face difficulties. If the percent of polymorphic loci is a rather reliable indicator, the value of the gene flow on the graph looks not always realistic. E.g., curiously, all the types of eucalyptus, apart from Eucaliptus pauciflora, proved to have such a low Nm. These are forest forming species of trees with large populations and long-range transport of blossom dust by wind, birds, and bats. By the value of the gene flow, they must be closer to coniferous trees than to autopollinated plants.

Only the gene flow of Е. pauciflora – Nm = 5.6 lends credence. For the rare E. сaesia, few in numbers, the gene flow Nm = 0.0001 looks realistic as well. However, hardly it is adequate in the widespread E.delegatensii (Nm = 0.606), and E.obliqua (Nm = 0.438). It can be assumed that the real number of interpopulation crossings in them is close to that in E. pauciflora, rather than E. caesia. Indeed, analyzing the initial work shows that the gene flow in Е. pauciflora was studied at the average distance of 5 km, whereas that in E.obliqua – at the distance of 400 km (!) It is apparent that the last one on the graph must be positioned close to Е. pauciflora [21; 22; 23].

We have encountered difficulties, arising from the methodological differences when collecting the initial data. The concept of “dynamic conservation of genes” is a possible solution.

It emphasizes maintaining evolutionary processes in the populations of perennial plants to protect their potential for a continuous adaptation (mostly, in situ, but considering dynamically developing ex-situ populations*). Gene conservation unit (GCU) relates to the population of a certain geographic location, selected as one that represents a high potential for adaptive evolution due to maintaining stability of population and metapopulation* [24]. The suggested approach is based on the assumption that within a species, gene conservation units constitute a series of differentiated metapopulations.

In real populations, a phenotype, rather than a locus as such, serves as a selection object. It combines an entire set of “co-selected” variants of numerous loci [25]. Therefore, our S emp constitutes a current result of the competition between several phenotypes. It is clear that S emp is an averaged indicator of selection power for the number of phenotypes and for even greater number of loci. However, it does not provide us with an overall picture of intrapopulation status of the species.

When an intense stabilizing selection occurs, the species is in a steady state, while with a limited selection it is in the midst of crisis, and an elementary evolutionary act, i.e., elimination of one or several biotypes, is highly probable. Analyzing Semp enables us to present the microevolutionary status of a species as a whole, though if we want to evaluate the potential destiny of certain intraspecific forms, we will need another approach.

Assume that we are aware of some endangered species being in a far-from-equilibrium state. A disruptive selection takes place here, threatening to transform such a species into several novel ones. Or, conversely, an intense stabilizing selection eliminates all the variants, except for some most adapted one, what, in terms of the major dogma of conservation genetics, also decreases adaptiveness.

However, our goal is to conserve the species. Do we need human intervention to do it? An F_ST_-statistics-based method is unable to answer this question. To forecast the destiny of a specific intraspecific form, whether it is gene or phene, a comparative stability of specific competing forms inside the gene pool will be of our interest, rather than an averaged indicator of stability of the entire gene pool for a number of loci. In terms of conservation of diversity, not changes in the occurrence, but the possibility of an elementary evolutionary act, namely, the elimination of one of forms must be considered. We will need the probability theory for this purpose.

## Methods of evaluating the effect of selection based on the probability theory

The situation, when there are several forms in a population, is called a balanced polymorphism. Hence, in a fully or almost fully isolated population, a possibility of elementary selection act must be defined by the degree, to which the polymorphism is balanced (in terms of stability of the existence of all its forms), and how probable the fact is that, in some conditions the balance is disrupted (extinction of one or several forms). Fluctuations of occurrence, sometimes leading to elimination, are likely to be caused by some environmental factors, the scenario of changing which in a specific case can be unknown to us or non-existent at all. The use of a probabilistic approach is suggested in such a situation.

Since Darwin’s days, the common knowledge lies in the concept of an evolutionary act as a result of losing stability in the ancestral form. However, if the initial form has lost its stability, it means that several forms are observed in the population, each of which is characterized by its stability. In this case, the differences in the adaptiveness of forms must be manifested as the differences in stability.

Hence, as a main parameter, describing the future of a form, it is more suitable to consider the form stability, closely related to the overall impact of environmental factors, as well as the lifetime of a form, rather than the adaptiveness or selection coefficient. In recent years, fairly complex models of computing a survival rate of the population of organisms have been created [26], where numerous parameters have been used. Studying heterozygosity, chromosomes, reproductive success, and other similarly labor-intensive research will be required for them. Unfortunately, the authors of the models do not consider the stability of a form within the population.

In the general case, the form stability is best judged by the behavior of the form itself, i.e., by the characteristics of fluctuations in its occurrence. At first sight, the solution seems simple: the greater the range of fluctuations in the form occurrence, the lower the form stability. If the fluctuations in the forms’ occurrence had been defined only by random combinations of environmental factors, the stochastic model would have provided a rather accurate evaluation of the probability of achieving any state of the system, including the extinction moment (reaching the zero number of one of the forms).

However, it should be remembered that against a backdrop of the stochastic process, a directed tendency must also manifest itself, determined by unequal adaptivenesses of competing forms. In other words, one is forced to apply a stochastic model to a process that is not quite stochastic. Hence, to analyze the empiric data, we have proposed to model the observed fluctuations, as a random process, and to consider the tendency as a systematic error, which value can be estimated, using an auxiliary model of linear regression.

We have found that a transformed birth-death model, thoroughly described by D. Goodman [27], meets our requirements to the stochastic model. This model has established a functional dependence of the actual (as of the moment of measurement) absolute number of population on the mathematical expectation of its lifetime. Thus, if during the monitoring of the population number, we have seen its extinction, it can be verified, how probable the deviation is of the observed lifetime of the population from the mathematical expectation, “predicted” by the model.

In our case, the model is applied not to the population as a whole, but to the individual forms, presented therein, and “good” predictions of the lifetime, obtained for several forms, which became extinct (or almost extinct) before the researcher’s eyes, will indicate the good quality of the model.

The model formula is as follows:$T(N)=\sum_{x=1}^{N}\sum_{y=x}^{N_{m}}\frac{2}{y(\mathrm{yV}(y)-r(y))}\prod_{z=x}^{y-1}\frac{\mathrm{zV}(z)+r(z)}{\mathrm{zV}(z)-r(z)}$, where T(N) – mathematical expectation of the population lifetime;

N – number of population at the “initial” moment in time, from which a lifetime is computed;

$N_{m}$ – maximum possible – maximum number of population (it is usually considerably higher than the ecological capacity of the habitat);

r(n) – mathematical expectation of the specific (per individual) rate of growth at the population number n;

V(n) – dispersion of specific (per individual) rate of growth at the population number n

It should be noted that as envisioned by the model for all $1\leq n\leq N_{m}$, expression V(n)>r(n) must be met. Otherwise, the values yV(y)−r(y) and zV(z)−r(z) can become zero or get close to it, what will result in an artifact. Hence, if the growth rate and its dispersion are constant (e.g., averaged) values $r(n)=\mathrm{const}=\overset{ˉ}{r}$ and $V(n)=\mathrm{const}=\overset{ˉ}{V}$ (it has been this case which we will consider below), expression $\left|\;\overset{ˉ}{r}\right|<<\;\overset{ˉ}{V}$ must be met, to be on the safe side.

The model was employed by its creators to evaluate the lifetime of island populations of northern mammals in the upper belts of the Rocky Mountains in USA, where they are the Pleistocene relicts. In many cases, the result has proved to be rather close to the time of their individualization, known by the paleoclimatic information [28]. Thus, it would be safe to assume that the model functions properly.

A mathematical expectation calculated using the model, constitutes a probable expected number of generations, over which the number of the studied form will become zero with a 95% probability. We have proposed to employ this indicator to have a comparative characteristic of the stability of forms in the polymorphous population.

## The results of applying a stochastic model

Out attempt to use the model for analyzing the data about emergence and extinction of stem rust races in the territory of USA [29], has demonstrated that the lifetime for a number of races was truly close to the mathematical expectation predicted by the model. As expected, the prediction has fallen short of expectations for those races, where a tendency for direction-oriented selection has been observed inside the occurrence fluctuations, traced using a linear regression formula. Undoubtedly, in the nearest future, a model will be developed with allowance for a direction-oriented trend.

**Figure 3.**
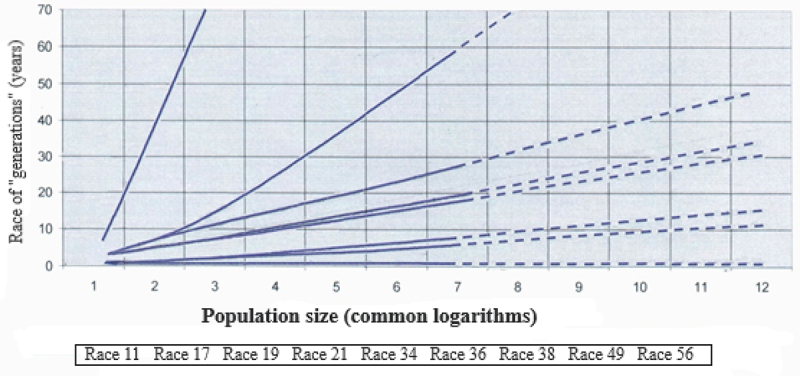
Maximum expectancies of stem rust races existence (Yuriev A.I., Nikulin V.A. [29]).

Of a particular interest was the fact that the expected lifetime of stem rust races, existing in one population, differs several hundred-fold (Figure 3). So enormous the power of selection proved to be. Since stem rust is a wheat pest, humans have arduously brought it under control, regularly introducing varieties, resistant to it. This is the case of very rapid evolution of a parasite following a rapidly evolving host, what is likely to cause such an intense selection.

Over a period of observations, two of the three races, defined as “unstable” ones, became extinct; and only one race of the four, defined as “the medium stable races”, disappeared. No races became extinct among the two races with the high expected lifetime.

## Discussion and interpretation of the results of applying a stochastic model

It stands to reason that the mathematical expectation of the lifetime is not a prediction of the real lifespan of a class of individuals. This is a period of time, over which the number of the class of individuals will, with a probability of 95%, reach the zero value. In actual life, the studied class can become extinct sooner or later. Such a probabilistic index is inadequate to make accurate predictions, though it is rather suitable for a comparative evaluation of the current state of the competing classes of individuals.

Over 15 years of observations, most races of stem rust, specified by this indicator as “low stability” races, really became extinct. This indicates that it is possible to predict forthcoming extinction with a rather high probability. Undoubtedly, the accuracy of predicting can be enhanced through corrections for nonrandom, direction-oriented trends in occurrence fluctuations.

Anyway, a ratio between mathematical expectations of the lifetimes of individuals’ classes within the population comprehensively characterizes the power of selection and the future outcome of a competitive struggle between them.

## Conclusion

The F_ST_-statistics-based method therefore allows for distinguishing among rare and protected species those, which require genetic monitoring first. The same method can be used to distinguish among common or agriculturally important species those subjected to both intense disruptive, and intense stabilizing selection.

Then, a method can be applied to these species, which is based on the probability theory and allows for predicting the outcome of a competitive struggle of the classes of individuals within the population.

Aravanopoulos suggested three indicators for the genetic monitoring, based on gene-ecological approach: natural selection, genetic drift, and gene flow system. They are evaluated based on three demographic (age- and size-related distribution, reproductive suitability, regeneration number) and four genetic (efficient population size, allele abundance, hidden genetic potential, outcrossing/ actual speed of in-breeding) parameters [9].

The methods for evaluating selection, we have suggested, rely on the data about the efficient size of population, allele abundance, and incidence of the classes of individuals within the population, what simultaneously characterizes a hidden genetic potential as well. Thus, the indicators, proposed by Aravanopoulos, harmonize well with our approach. Certainly, to determine the reproductive ability and age-related distribution is also useful, since it allows for presenting the occurring processes in detail.

Our methods enable assessment of both vulnerability of the genetic state of the species as a whole, and stability of an individual gene or a combination of genes. However, to solve the second problem we must have the data of observations over at least 5–7 generations of the studied species. Such research studies are rare with the present-day organization of science. E.g., L. Laikre with the colleagues from 775 scientific publications, devoted to the population genetics of Swedish animals and plants, had found only 4, adequately representing temporal changes of the data over a number of generations. All the four works related to the brown trout, a commercial salmonid fish [8].

Now, upon the “genetic monitoring programme” request, the GOOGLE SCHOLAR database provides plenty of works, though the vast majority of them, as in 2008, has still devoted to one-time studies of the genetic structure of an object. Hence, as Laikre reasonably notes, these are not the programs of monitoring. However, such a one-time research has often incorporated computation of the allele abundance and F_ST_ indicators. It enables the use of the above-described method for determination of the value of disruptive selection and assessment of the need for long-term observations, with regard to the studied species.

Perhaps, the approach accepted in many funds to awarding the grants fails to facilitate extensive (“temporal”) genetic studies. Long-standing programs of observations, e.g., the Records of Nature, approved in the nature reserves of Russia, have also been free from regular genetic studies of any species.

Hence, the special funds are needed for financing long-term genetic studies. They can be private or national, depending on the specifics of a particular country.

It would be logical if every country had a national list of genetically vulnerable species, as a supplement to its Red Book. Such a list can be made relying on only one-time genetic study of each species, using the above evaluation method of selection based on f-statistics, and will not require huge expenses. It is precisely the revealed genetically vulnerable species that must become a focus of the special-purpose programs of regular genetic monitoring.

## Conclusions

1. The problem of distinguishing the species subjected to abnormal disruptive or stabilizing selection, can be solved using the f-statistics-based method. The problem of analyzing the results of extensive genetic monitoring can be solved using a method based on the probability theory.
2. An F_ST_-statistics-based method can be finalized and revised using the existing genetic study programmes, allowing for determination of the value of allele abundance and gene flow.
3. The value of disruptive/stabilizing natural selection is a parameter, which specifies the assessment of the degree of threat to a species, obtained through the traditional Fst-statistics. It must be considered together with the traditional indicators of genetic monitoring.
4. The expected lifetime of a gene/phene in the population is a parameter that makes it possible to compare the classes of individuals, existing in the population, by stability, to predict the probable outcome of a competitive struggle, and to plan based thereupon the measures to conserve rare or economically valuable genotypes.
5. The species subjected to an intense disruptive/stabilizing selection must become the object of regular genetic monitoring once in every generation. To finance such works, the special-purpose national or private funds must be created.
6. The advantage of assessing the value of natural selection over classic F_ST_-statistics lies in the opportunity for at least approximate prediction of future changes in the population.
